# Issue of Data Imbalance on Low Birthweight Baby Outcomes Prediction and Associated Risk Factors Identification: Establishment of Benchmarking Key Machine Learning Models With Data Rebalancing Strategies

**DOI:** 10.2196/44081

**Published:** 2023-05-31

**Authors:** Yang Ren, Dezhi Wu, Yan Tong, Ana López-DeFede, Sarah Gareau

**Affiliations:** 1 Department of Computer Science University of South Carolina Columbia, SC United States; 2 Department of Integrated Information Technology University of South Carolina Columbia, SC United States; 3 The Institute of Families in Society University of South Carolina Columbia, SC United States

**Keywords:** low birthweight, machine learning, risk factor, benchmark, data rebalance

## Abstract

**Background:**

Low birthweight (LBW) is a leading cause of neonatal mortality in the United States and a major causative factor of adverse health effects in newborns. Identifying high-risk patients early in prenatal care is crucial to preventing adverse outcomes. Previous studies have proposed various machine learning (ML) models for LBW prediction task, but they were limited by small and imbalanced data sets. Some authors attempted to address this through different data rebalancing methods. However, most of their reported performances did not reflect the models’ actual performance in real-life scenarios. To date, few studies have successfully benchmarked the performance of ML models in maternal health; thus, it is critical to establish benchmarks to advance ML use to subsequently improve birth outcomes.

**Objective:**

This study aimed to establish several key benchmarking ML models to predict LBW and systematically apply different rebalancing optimization methods to a large-scale and extremely imbalanced all-payer hospital record data set that connects mother and baby data at a state level in the United States. We also performed feature importance analysis to identify the most contributing features in the LBW classification task, which can aid in targeted intervention.

**Methods:**

Our large data set consisted of 266,687 birth records across 6 years, and 8.63% (n=23,019) of records were labeled as LBW. To set up benchmarking ML models to predict LBW, we applied 7 classic ML models (ie, logistic regression, naive Bayes, random forest, extreme gradient boosting, adaptive boosting, multilayer perceptron, and sequential artificial neural network) while using 4 different data rebalancing methods: random undersampling, random oversampling, synthetic minority oversampling technique, and weight rebalancing. Owing to ethical considerations, in addition to ML evaluation metrics, we primarily used recall to evaluate model performance, indicating the number of correctly predicted LBW cases out of all actual LBW cases, as false negative health care outcomes could be fatal. We further analyzed feature importance to explore the degree to which each feature contributed to ML model prediction among our best-performing models.

**Results:**

We found that extreme gradient boosting achieved the highest recall score—0.70—using the weight rebalancing method. Our results showed that various data rebalancing methods improved the prediction performance of the LBW group substantially. From the feature importance analysis, maternal race, age, payment source, sum of predelivery emergency department and inpatient hospitalizations, predelivery disease profile, and different social vulnerability index components were important risk factors associated with LBW.

**Conclusions:**

Our findings establish useful ML benchmarks to improve birth outcomes in the maternal health domain. They are informative to identify the minority class (ie, LBW) based on an extremely imbalanced data set, which may guide the development of personalized LBW early prevention, clinical interventions, and statewide maternal and infant health policy changes.

## Introduction

### Background

The recent Centers for Disease Control and Prevention (CDC) annual report indicates that babies with low birthweight (LBW), defined as an infant weight of <2500 g by the World Health Organization [1], accounted for 8.24% of all births in the United States in 2020 [2]. Alarmingly, LBW was the second leading cause of neonatal death in the United States after congenital malformations in 2019 and 2020 [3]. Moreover, babies with LBW have a higher risk of short- and long-term adverse health effects than those with a normal birthweight, such as heart and lung complications and associated chronic diseases [4]. Tremendous financial burdens have also been imposed on the families of the babies with LBW and health care payers [5]. Previous studies have found that maternal demographics [6], preexisting health conditions [7], social determinants [8], and prenatal care level [9] are associated with LBW. Thus, precisely identifying which pregnant patients may be at the greatest risk of having a baby with LBW in the preconception or early pregnancy stages is critical to save neonatal lives and reduce potentially avoidable medical expenditures through direct clinical and health policy interventions. Our benchmarking models and feature importance analysis results have the potential to aid in this initial clinical screening and identification of high-risk birthing people and the development of policies that improve health care quality and invest in communities of most opportunity.

In recent years, with the exponential growth in the quantity and dimension of health care data, machine learning (ML) methods have been introduced to handle complex and high-dimensional data [10,11]. Numerous studies have shown that ML algorithms achieved good performance on target prediction [12,13], outcome estimation [14], and risk factor analysis [15,16] in the field of precision medicine. Although ML is used as a new computational method to explore various health problems, the main challenge of ML applications in the health domain is the common issue of imbalanced data that disproportionately focus on the health of minority groups in ways that may generate adverse events in the whole population [17]. An imbalanced class distribution poses a challenge to the performance of ML models. These models trained on imbalanced data sets can produce misleading results for the intended prediction tasks. These models often yield suboptimal classification results and may erroneously treat rare minority examples as noise [18]. In addition, the use of global performance metrics such as prediction accuracy to guide the learning process can create a bias toward the majority class, leading to a lack of awareness of rare events even if the prediction model achieves high overall accuracy [18,19]. In the prenatal health care field, ML models face the same challenges with imbalanced data sets for correct predictions because of the lower frequency of poor outcomes compared with normal outcomes. Hence, identifying key benchmarks is crucial to guiding proper ML use in perinatal care, maternal health, and other health domains. This is a considerable knowledge gap that we aimed to fill in this study.

Current documented studies on ML use in perinatal care and maternal health are scarce. Few previous LBW prediction studies have achieved good performance on imbalanced small data sets with limited features (ie, variables) [20,21] or rebalanced training and testing sets [22,23]. However, the ML results from these studies could be problematic, misleading, and not generalizable as they did not disclose their LBW distributions and methods of effectively handling any data imbalance issues. In this study, we aimed to conduct a more systematic and accurate analysis of LBW cases in an extremely imbalanced data set based on a large delivery population. The data records were classified into 2 groups based on birth outcomes—LBW and non-LBW—and the size of the LBW group was less than a tenth of that of the non-LBW group. The unequal distribution of these 2 groups caused a substantial imbalance in the data, which is a common issue in the perinatal health domain because of the incidence of LBW. In this study, we evaluated the performance of 7 classic ML models—logistic regression (LR), naive Bayes (NB), random forest (RF), extreme gradient boosting (XGBoost), adaptive boosting (AdaBoost), multilayer perceptron (MLP), and sequential artificial neural network (ANN)—on the LBW prediction task for a large-scale data set that linked 266,687 birth records with uniform billing (UB) hospital records and Medicaid eligibility from the first quarter of 2015 to the first quarter of 2021 in a southeast state in the United States. In addition to the target variable, LBW, our data set contained 155 other variables. In this study, we applied 4 different data rebalancing methods to each of the baseline models: random undersampling, random oversampling, synthetic minority oversampling technique (SMOTE) [24], and class weight adjustment. We conducted extensive experiments to obtain benchmarking results using the 7 classic ML models on both the original imbalanced data set and different rebalanced data sets; as a result, both scholars and health care practitioners can use our benchmarks to serve as a baseline for their LBW classifications, especially those who have access to large-scale imbalanced data sets. We also analyzed the LBW risk factors to determine the common risk factors that were identified by the best performing models using the most effective rebalancing method. The risk factor analysis results could provide suggestions for initial clinical screening criteria.

### Related Work

In this section, we conducted a comprehensive literature review in the area of perinatal health studies that applied ML methods using claims data and identified a limited number of studies predicting LBW. To garner a more holistic understanding of the field of ML-based LBW prediction–related studies, Table 1 compares the study data sets, applied methods, prediction models, best performance, and risk factors. To clearly compare their input features, we further grouped them into four categories: (1) physical factors, including basic maternal information such as age, race, height, weight, and BMI; (2) social factors, including education level, job category, wealth index, and residence; (3) medical factors, ranging from different indicators of medical history such as diabetes and hypertension to laboratory reports, including indicators of different laboratory tests; and (4) nutritional factors, representing maternal nutrition intake records.

Table 1 summarizes the reviewed literature into 3 categories based on their data size and imbalance issues. The first group of studies used a small data set consisting of <1000 records [20,21,25,26]. In this group, 50% (2/4) of these studies reported the percentage of LBW cases, which highlights the presence of data imbalance issues [20,21]. However, none of these studies addressed the data imbalance issue or implemented rebalancing methods. Although these studies achieved remarkable ML model performance on a small imbalanced data set, the results could be misleading and biased toward the majority class (ie, non-LBW) owing to the data imbalance issue causing them to learn based on the error rate without considering the class distribution. The studies in the second group used larger imbalanced data sets but still did not apply any rebalancing methods to their imbalanced data sets [22,23,27-29,34]. Their high accuracy and low area under the receiver operating characteristic curve (AUROC) scores revealed that misleading performance remains a persistent issue [33]. The third group comprises studies that used data rebalancing techniques for their imbalanced data sets [30-32]. However, these studies only applied a single data rebalancing method to multiple ML models, either oversampling [30] or SMOTE [31,32]. Therefore, it was still unclear which data rebalancing method was optimal for addressing data imbalance issues in LBW prediction tasks across different ML models.

Another major issue for studies that used ML models was their insufficient sample size with a single population. Their small data sets did not adequately represent the actual demographic distribution of the data in the population. The 2 primary data sources of the previous studies were hospital records and demographic surveys. The studies using survey data that included the parents’ basic information and the baby’s weight may have reached a larger sample size. However, no features from the patient hospital visits were included, such as medical factors, which are important indicators for determining birth outcomes [6]. Regarding the studies that used hospital records, their data features were richer than the survey data, with common limitations of very small sample sizes and poor data quality because of data access issues and policy restrictions.

We conducted a comparison of features across all studies and analyzed the details of the data rebalancing methods for the applicable studies. Through this comparison, we identified instances of data leakage and reproducibility issues. To further justify our recognized concerns, we referenced a previous study that comprehensively examined and synthesized the problems surrounding data leakage and reproducibility in ML-based scientific research [35], and its findings provided a theoretical basis for our literature review and comparison.

According to the “Summary of features” column in Table 1, certain studies used the baby’s features or the features only available after the delivery admission, such as baby sex, baby delivery method, and gestational age [21,28,30-32]. Using such features in a model could potentially result in data leakage as the model would be unable to access this information while making predictions about a new patient’s outcome. This issue can be identified as a model using nonlegitimate features [35].

We then compared the data rebalancing process in the studies that incorporated data rebalancing methods [30-32]. Notably, all these studies [30-32] applied their data rebalancing methods to the entire data set before splitting the training and testing sets, leading to inaccurate and misleading prediction results as the testing set could not represent the original data distribution after being rebalanced. In contrast, this issue could lead to leakage because of a lack of clean separation of training and test data sets [35]. For example, oversampling was performed before dividing the data into training and testing sets [30], resulting in data sets that would not be perfectly separated as the oversampled data generated from the training set would also be present in the test set.

To overcome the aforementioned limitation of small data size, we applied a large-scale data set that combined vital statistics birth records with UB-04 hospital records and Medicaid eligibility data. This comprehensive data set contained 266,687 birth records and included 158 features. To prevent the legitimate feature issue, we only selected the predelivery variables as the models’ input features. For the imbalanced data set, to improve the model performance and identify the best data rebalancing solution for our data set, we applied 4 data rebalancing methods to the training set only and used the original testing set to compare the prediction performance of 7 ML models for the LBW prediction task: LR, NB, RF, XGBoost, AdaBoost, MLP, and sequential ANN. For the performance evaluation, we focused on reducing the error rate of predicting the LBW case as non-LBW, which could cause severe real-life consequences. Moreover, owing to the imbalanced data set, the accuracy could not adequately examine the performance of the minority group. Thus, we evaluated the prediction based on the AUROC score and monitored the recall score, which quantifies the number of actual LBW cases that are correctly predicted as LBW.

**Table 1:** Summary of low birthweight (LBW) prediction– and classification-related studies.

Study			Data size (LBW %)		Number of input features		Summary of features		Rebalancing method		Prediction model		Best performance^a^		Identified risk factors
**Previous studies with small sample sizes**															
	Yarlapati et al [25], 2017	101 (not reported)		18		Mothers’ predelivery factors: physical, social, medical, and nutritional		None		Bayes minimum error rate		Accuracy: 96.77%; recall: 1.0; AUROC^b^: 0.93		Mothers’ living community, age, and weight	
	Senthilkumar and Paulraj [20], 2015	189 (31.22)		11		Mothers’ predelivery factors: physical		None		LR^c^, NB^d^, RF^e^, SVM^f^, NN^g^, and CT^h^		CT—accuracy: 89.95%; recall: 0.98; AUROC: 0.94		Last weight before pregnancy, mother’s age, number of physician visits during the first trimester, and number of previous premature labors	
	Ahmadi et al [21]^i^, 2017	600 (9.5)		17		Baby: sex and delivery method; mothers’ predelivery factors: physical, social, medical, and nutritional		None		RF and LR		RF—accuracy: 95%; recall: 0.72; AUROC: 0.89		Pregnancy age, BMI, and mother’s age	
	Desiani et al [26], 2019	219 (not reported)		6		Mothers’ predelivery factors: physical, social, and medical		None		NB		Accuracy (for LBW group): 81.25%; recall (for LBW group): 0.72; AUROC: not reported		None	
**Previous studies with larger sample sizes without using any rebalancing methods**															
	Akmal and Razmy [27], 2020	2702 (not reported)		12		Mothers’ predelivery factors: physical and medical		None		BF^j^ tree, C4.5, RF, random tree, REP^k^ tree, and logistic model tree		C4.5—accuracy: 79.23%; recall: 1.0; AUROC: not reported		None	
	Islam et al [28]^i^, 2022	2351 (16.2)		17		Baby: sex, singleton, and delivery method; mothers’ predelivery factors: physical, social, and medical		None		LR and DT^l^		LR—accuracy: 87.6%; recall: 1.0; AUROC: 0.59		None	
	Borson et al [22], 2020	4498 (not reported)		9		Mothers’ predelivery factors: physical and social		None		LR, NB, KNN^m^, RF, SVM, and MLP^n^		SVM and MLP—accuracy: 81.67%; recall: 0.82; AUROC: not reported		None	
	Faruk and Cahyono [23], 2018	12,055 (<10)		8		Mothers’ predelivery factors: physical and social; fathers’ factors: social		None		LR and RF		RF—accuracy: 93%; recall: unknown; AUROC: 0.51		Top 3 features: mother’s age, time zone, and wealth index	
	Eliyati et al [29], 2019	12,055 (<10)		8		Mothers’ predelivery factors: physical and social; fathers’ factors: social		None		SVM		Accuracy: 92.9%; recall: not reported; AUROC: 0.56		None	
**Previous studies using rebalancing methods**															
	Loreto et al [30]^i^, 2019	2328 (13.45)		8		Baby: sex; mothers’ predelivery factors: physical and medical; mothers’ non-predelivery factor: gestational age		Oversampling		AdaBoost^o^, CT, KNN, NB, RF, and SVM		AdaBoost—accuracy: 98%; recall: 0.91; AUROC: not reported		None	
	Bekele [31]^i^, 2022	2110 (10)		25		Baby: sex and delivery method; mothers’ predelivery factors: physical, social, and medical		SMOTE^p^		LR, DT, NB, KNN, RF, SVM, gradient boosting, and XGBoost^q^		RF—accuracy: 91.6%; recall: 0.92; AUROC: 0.97		Gender of the child, marriage to birth interval, mother’s occupation, and mother’s age	
	Khan et al [32]^i^, 2022	821 (10.84)		88		Mothers’ predelivery factors: physical, social, and medical; fathers’ factors: social; mothers’ non-predelivery factor: gestational age		SMOTE		Zero, KNN, NB, kStar, MLP, random tree, SVM, AdaBoost, LR, RF, OneR, stacking, stack, DT, and bagging		LR—accuracy: 90.24%; recall: 0.90 (accuracy on LBW: 33%)		Diabetes, gestational age, and hypertension	

^a^The performance measurements are explained in Multimedia Appendix 1 [33].

^b^AUROC: area under the receiver operating characteristic curve.

^c^LR: logistic regression.

^d^NB: naive Bayes.

^e^RF: random forest.

^f^SVM: support vector machine.

^g^NN: neural network.

^h^CT: classification tree.

^i^The study applied features from the baby side or the mothers’ non-predelivery features.

^j^BF: best first.

^k^REP: reduced error pruning.

^l^DT: decision tree.

^m^KNN: k-nearest neighbors.

^n^MLP: multilayer perceptron.

^o^AdaBoost: adaptive boosting.

^p^SMOTE: synthetic minority oversampling technique.

^q^XGBoost: extreme gradient boosting.

## Methods

### Overview

**Figure 1:**
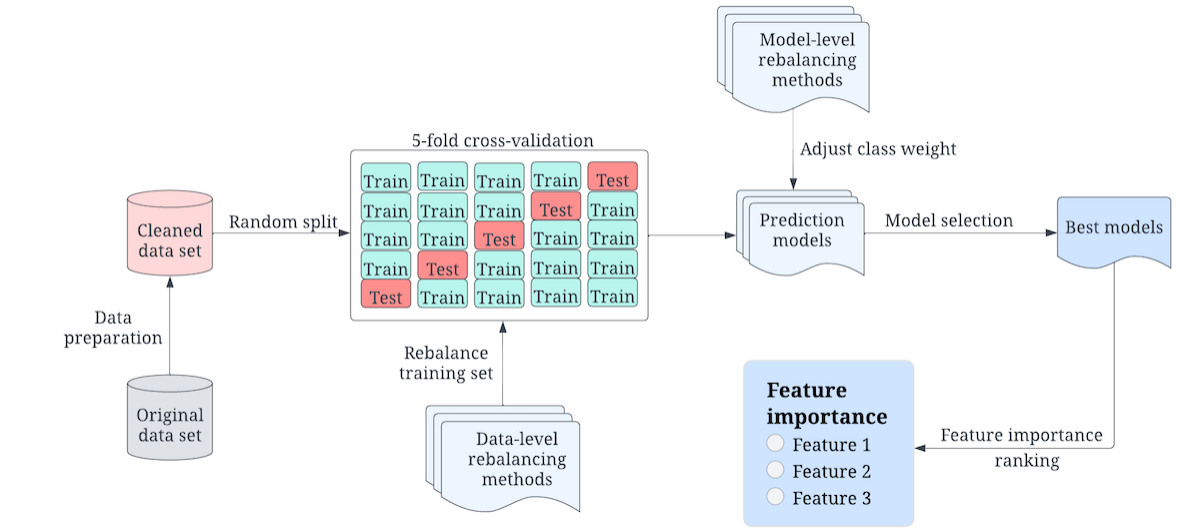
High-level structure of low birthweight prediction study design.

This study applied 7 ML models and 4 distinct rebalancing methods to predict the LBW group and analyze related risk factors. The process was carried out in multiple stages, as shown in Figure 1, which included data preparation, development of LBW prediction models, model evaluation and selection, and associated risk factor analysis. This section provides a detailed description of each stage.

### Study Data

The findings from this retrospective cohort study are based on 266,687 birth records from the first quarter of 2015 to the first quarter of 2021 from a large health care system in a southeast state of the United States. All patients’ personal information was deidentified and not disclosed based on the Health Insurance Portability and Accountability Act privacy and security policy. The data set inclusion criteria were based on maternal inpatient hospitalization or emergency department (ED) visit claims that included any International Classification of Diseases, 10th Revision (ICD-10), or Medicare Severity Diagnosis Related Groups codes for delivery diagnosis or procedure. The exclusion criteria were abortion, false labor, threatened abortion, and missing linkage of claim record with the birth record.

As the main target variable of this study, birthweight was categorized into 3 distinct levels by our data provider: extreme LBW (ELBW; <1500 g), moderate LBW (1500-2499 g), and not LBW (≥2500 g) [1]. Owing to the population of very LBW being <1.45% of the total number of records, we combined the ELBW and moderate LBW into 1 category (ie, LBW). Thus, the target variable in this study has 2 categories: LBW (≤2499 g) and not LBW (≥2500 g), which is the national standard definition [2]. We also categorized the target variable into ELBW and non-ELBW (≥1500 g) groups to further validate the models we developed for the LBW prediction task.

In addition to the target variable, each record had 155 variables from three periods: (1) the 12 months before delivery, (2) during delivery, and (3) the 12 months after delivery. As our focus was on identifying factors that could be part of early prenatal screening for high LBW risk, we only selected the variables from 12 months before delivery as the input variables to develop our ML prediction models in this study. Previous studies have shown that hospital information [36,37], patient demographics [38,39], patients’ preexisting conditions [40,41], and social determinants [42] are closely related to LBW outcomes and could be regarded as the underlying risk factors for LBW. In this study, the input variables encompassed 2 hospital-related factors (ie, perinatal care level and hospital baby-friendly status) and patients’ demographic information, including age, race, payment source, zip code, first pregnancy status, number of fetuses delivered, and number of previous deliveries. The study also considered preexisting maternal conditions, including cardiovascular disease, hypertension, diabetes, obesity, substance abuse, mental health, opioid use, gestational diabetes, pre-eclampsia, intensive care unit stay, sum of inpatient hospitalizations, sum of ED visits, and composite condition profiles (chronic and behavioral health). Furthermore, the input features included the CDC Agency for Toxic Substances and Disease Registry social vulnerability index (SVI) indicators [43]. We provide a detailed breakdown of the distribution of these features in the *Results* section.

### Ethics Approval and Data Compliance

This study was approved by the institutional review board of the University of South Carolina (approval Pro00100005). For the research data set used in this project, all data were deidentified and securely stored in our Health Insurance Portability and Accountability Act–compliant research server, and only authorized researchers could access them.

### Data Preparation

Before proceeding with data analysis, we cleaned the raw data that our data provider initially processed. At this stage, we focused on identifying records with mislabeling, missing values, or inconsistent scale values. We first dropped the records with missing values or unknown values in the variables related to patients’ demographics and birth outcomes to avoid invalid records and improve data quality. Next, to address all other missing values that existed in the preexisting condition variables, we merged them into the “No/Unknown” group [44]. In doing so, we preserved as much information as possible while avoiding the inducing bias found in other approaches (eg, complete case analysis and imputation) that mishandle missing value prediction [45]. As we focused on the records from a single US state, we also removed the records in which the mother’s region was marked as out of state. As a result, the cleaned data set size was reduced from 266,687 to 255,467 (95.79%) records.

To effectively identify which pregnant patients would be at the highest risk of delivering a baby with LBW, we removed the features available only at the delivery phase or after delivery from our data set. The removed features included delivery method, gestational age, patients’ disease indicators when admitted to labor or within 12 months after delivery, and different indicators for neonatal care. After feature selection, the ML models’ input features only included the features available before delivery. We then re-encoded the categorical variables into numbers to facilitate the use of ML models. When we analyzed the feature importance to effectively (1) differentiate each subcategory and (2) improve the expressiveness of input variables with multiple categories, we also applied the one-hot encoding method to convert the variables with multiple categories into multiple binary variables [46]. For example, the variable of race was converted into 4 binary variables: non-Hispanic White, non-Hispanic Black, Hispanic, and other non-Hispanic races. For the continuous variables in our data set, we normalized them into the same scale between 0 and 1 to speed up learning for faster convergence in the ML model training process [47]. The final cleaned data set included 9.01% (23,019/255,467) of LBW records and 90.99% (232,448/255,467) of non-LBW records with 54 variables. Of the 23,019 LBW records, there were 3708 (16.11%) cases of ELBW.

### ML Models

#### Overview

In this study, we developed 7 models for the LBW and ELBW prediction tasks based on different classic ML classification algorithms that are widely used to solve classification problems in different domains, especially for binary classification problems [48]. The applied ML algorithms included LR, NB, RF, XGBoost, AdaBoost, MLP, and sequential ANN. The most important ML classification algorithms can be categorized based on their nature, including decision tree–based algorithms, perceptron-based techniques, statistical learning algorithms, and support vector machine (SVM) [49,50]. RF, XGBoost, and AdaBoost are the most popular decision tree–based algorithms with ensemble methods to make the algorithms more robust than a single decision tree [51]. The perceptron-based techniques include different neural network models, and MLP and sequential ANN are powerful neural network classifiers that enable the learning of nonlinear functions for complex data. NB and LR are 2 typical statistical learning algorithms. The models applied in this study covered all the most important ML classification techniques except for SVM as SVM, a classic and powerful classifier, is unsuitable for the classification task on large-scale data owing to the training complexity [52].

In this study, we used the *scikit-learn* Python library [53] to develop our classification models. Our sequential ANN model was developed based on the Keras framework [54]. To evaluate the performance of a model and improve its ability to generalize to new, unseen data, we applied a 5-fold cross-validation to train and test the ML models. In each fold, we preserved the same proportion of the LBW class in the training and testing sets as observed in the full data set. To cross-compare the model performance on different data rebalancing methods, we used the default setting that was provided by the *scikit-learn* library for all the applied ML models. Using the default parameters setting when building classifiers is a common approach in ML as it provides a baseline for comparison. By using the default parameters, we could compare the performance of different classifiers without the influence of specific hyperparameters or other choices that could have been made differently for each classifier. In addition, using the default parameters helped ensure that the comparison between classifiers was fair and unbiased. Thus, hyperparameter tuning was not within the scope of this study, as discussed further in the *Limitations and Future Directions* section. In the following subsections, we describe details of the classification models and their architectures.

#### LR Model

LR is a classification method that is generally used to find the hyperplane or line that best separates positive samples from negative samples. From the provided data set, it learns a linear connection before introducing a nonlinearity in the form of the sigmoid function [55]. For our LR model, a maximum number of iterations of 100 was set for the limited-memory Broyden-Fletcher-Goldfarb-Shanno solver to converge, and the inverse of the regularization strength was set to 1.

#### NB Model

As a classic supervised ML algorithm, NB is widely used for structural data classification [56]. We built our NB classification model based on the Gaussian NB module from the *scikit-learn* Python library. On the basis of the Bayes theorem, NB considers each feature to contribute independently to the probability of the LBW class while ignoring the correlation among the features. Therefore, the NB model does not require extensive training data while requiring the training data to represent true data distributions.

#### RF Model

RF is a flexible, easy-to-use, and robust supervised ML algorithm. RF is based on the bagging algorithm and the ensemble learning technique. The RF model training process can be highly parallelized and, thus, efficient on large data sets with high-dimensional input [57]. The RF model could minimize the impact of outliers during classification owing to the characteristics of the decision tree structure. The outliers only affect the leaf nodes to which they belong and do not affect other leaf nodes. The parameters for the RF model were configured as follows: the forest contained 100 trees, the minimum number of samples required to split an internal node was 2, the minimum number of samples required to be at a leaf node was 1, and bootstrapped samples were used to build each tree in the forest.

#### XGBoost Model

XGBoost is an efficient supervised ML algorithm based on a distributed gradient-boosted decision tree [58]. It can provide reliable results and minimize the overfitting issue by using a parallel tree-boosting strategy. Furthermore, XGBoost can evaluate each feature’s importance using the importance score. In our XGBoost model, the parameters were set such that there were 100 trees and the maximum tree depth was 3. The learning rate was 0.3. In addition, 100% of the training data were used to build each tree.

#### AdaBoost Model

AdaBoost is an ensemble model with a typical boosting algorithm. In the AdaBoost model, the weight of each basic learner is adjusted based on the error rate of the results from the previous iteration. AdaBoost adds more weight to the samples mislabeled from the previous iterations in the following iteration [59]. Therefore, the model performance can be improved after a certain number of iterations. The base estimator in our AdaBoost model was a decision tree classifier with a maximum depth of 1, and the number of weak learners was 50. The learning rate was set to 1.0, which was used to scale each base estimator’s weight during the training iterations.

#### MLP Model

MLP is a deep learning network that applies the back propagation method during the training process. Back propagation is used to change the connection weights across layers based on the error rate in the predicted results for supervised MLP learning [60,61]. To develop the MLP model for our task, we set the range of hidden layer numbers from 1 to 5, and the neuron number was assigned from 100 to 500 with the same interval (100) to search for the best hidden layer structure. We then determined our MLP model structure with 2 hidden layers and 100 neurons in each hidden layer empirically. A rectified linear unit function was applied in the model, and the Adam solver for stochastic gradient descent was used. The learning rate was set to a constant value of 0.001. The training data were also shuffled before each epoch, and the maximum number of iterations was set to 200.

#### Sequential ANN

Sequential ANN models can learn representations of input features, which can be used to improve classification performance [54]. The sequential model allows for the creation of multilayer networks by stacking layers linearly, where the output from one layer is used as the input for the next layer [54]. Thus, the sequential ANN model is a powerful tool for building neural networks for classification tasks and is highly configurable by adding several types of layers, such as dense layers, convolutional layers, and recurrent layers, to suit specific problem requirements. The model can also be trained using various optimization algorithms such as stochastic gradient descent to minimize the loss between the predicted and actual class values. We used the dense approach in this model and the rectified linear unit activation function to construct 2 hidden layers. The input dimension of the model corresponds to the size of the features. The size of the output from the hidden layers was set to 32. After the hidden layers was an output layer with a sigmoid activation function and an output size of 1. Given that our task was a binary classification problem, we used Adam as the optimizer and binary cross-entropy as the loss function. There were 100 epochs, and the batch size was 2000 in each epoch.

### Data Rebalancing

#### Overview

In our data set, the LBW group was 9.01% (23,019/255,467), and the ELBW group was 1.45% (3708/255,467) after data cleaning, reflecting an extremely imbalanced data set. Accuracy is usually an intuitive and effective metric but not when evaluating the performance on imbalanced data. Considering a binary classification scenario in which 1% of the samples are positive, a classifier only needs to output all zeros to obtain an accuracy of 0.99, leading to meaningless classification results, which is a common problem in the public health domain. In addition to using different evaluation metrics, we tried different solutions at both the data and model levels to improve the ML model classification ability for predicting the minority group.

#### Data-Level Data Rebalancing Methods

**Figure 2:**
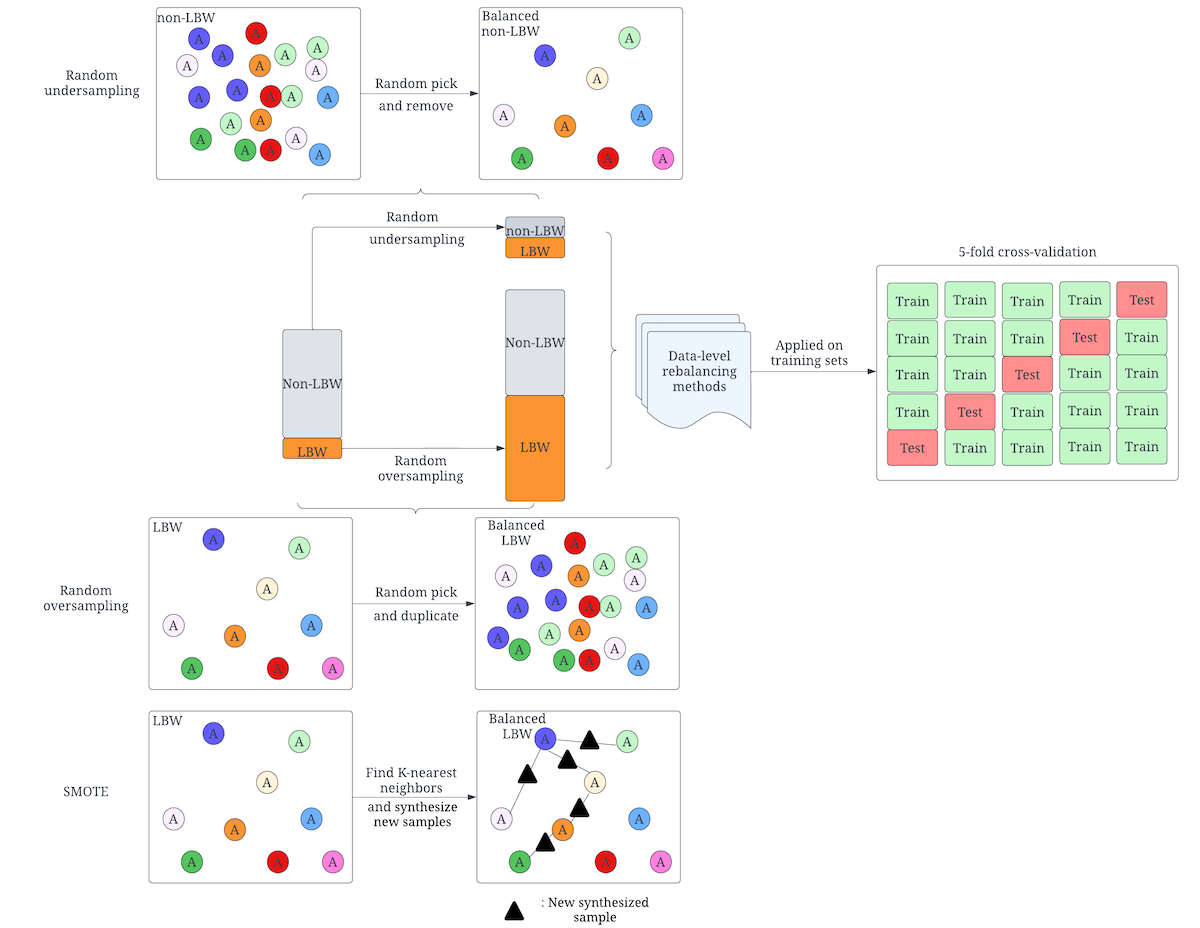
Detail-level structure of data rebalancing methods at the data level. LBW: low birthweight; SMOTE: synthetic minority oversampling technique.

To handle the imbalanced data problem at the data level, undersampling and oversampling are the 2 most common methods. As shown in Figure 2, the undersampling method was used to reduce the observations from the majority group until the distribution was balanced between the majority and minority groups [62]. The oversampling methods extend the size of a minority group to balance the distribution. The most common methods include random oversampling and SMOTE [24]. Random oversampling extends the minority size by randomly duplicating the minority samples, commonly leading to overfitting issues. SMOTE can alleviate this issue from random oversampling. For each sample in the minority class, the basic idea of SMOTE is to calculate the distance from it to all samples in the minority group and obtain the k-nearest neighbors. Then, a point between the sample and one of the k-nearest neighbors is randomly selected as the new synthesized sample of the minority class [63].

#### Model-Level Data Rebalancing Method

**Figure 3:**
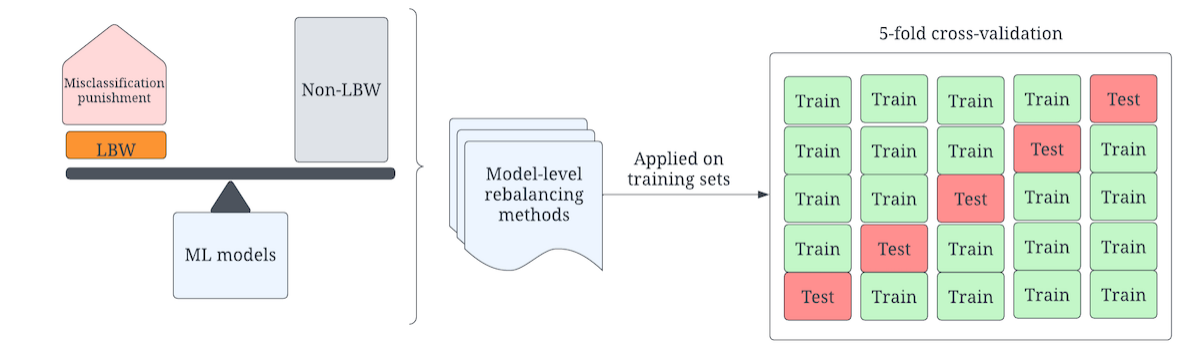
Data rebalancing at the model level. LBW: low birthweight; ML: machine learning.

At the model level, we addressed the data imbalance problem by directly adjusting the majority and minority class weights to modify the ML model training process, as shown in Figure 3. A higher weight was assigned to the minority group and a lower weight was assigned to the majority group to make the model accommodate the imbalanced distribution, and the adjusted class weight was based on the distribution (equation 1). These strategies force the model to penalize the misclassification made by the minority class in the training phase.

Specifically, the *scikit-learn* Python library has the built-in parameter “class_weight” for the LR, RF, XGBoost, and AdaBoost classifiers. The weight for each class is automatically assigned by the model to be inversely proportional to the corresponding frequency. The balanced weight for each class is calculated based on equation 1.


Class weight = total number of samples / (number of classes × class sample size) **(1)**

Then, for the LBW prediction on our data set, the LBW class weight was 5.55, and the non-LBW class weight was 0.55 under the balanced option. For the ELBW task, the weight of the ELBW was 34.45, and the non-ELBW class weight was 0.51. For the NB classifier, the imbalanced distribution was reflected in the prior probability of the class. Thus, we set the prior probability as 0.5 for each class to balance the groups. For the MLP classifier, we will adjust the class weight by modifying the weights in the loss function in future work.

### Evaluation Metrics

To evaluate the performance of each classification model, we used 5 different metrics widely used for measuring classification performance: *accuracy*, *precision*, *recall*, *F*_1_-score, and AUROC. We presented the mean score of each measurement across all folds in the cross-validation. In our LBW prediction task, the negative consequences would be more severe if LBW records were predicted as non-LBW compared with the issue of non-LBW records being predicted as LBW. Therefore, it was important to expect a high average *recall* score in our task, even compromising on a lower average *precision* score. Following this guideline, we focused on the average *recall* score when evaluating the model performance.

## Results

### Feature Understanding—Statistical Data Analysis

#### Overview

In this section, we analyze the distribution of each variable in the LBW group. We also applied the chi-square test and used the *P* value to determine whether there was a statistically significant association between the values of each variable and LBW. We applied the odds ratio (OR) and its CI to measure the strength and precision of the association between the features and the outcome [64]. More specifically, in our study, the OR was used to quantify the difference in the odds of LBW between the groups within the categorical variables. The CI provided a confidence range of values that is expected to contain the true OR. The width of the CI depends on the sample size and the variability of the data. A narrow CI indicates that the estimate of the OR is more precise, whereas a wide CI indicates that the estimate is less precise [64]. For the continuous variables, we show the CI of the mean value of the variables, which was used to provide important information about the uncertainty of the estimate of the population mean. The variables in our data set were divided into 3 categories: hospital-related variables, SVI indicators, and mother-related variables. The results for each category are discussed in the following subsections.

#### Hospital-Related Variables on LBW

This study included 23,019 LBW records, accounting for 9.01% (N=255,467) of the total delivery records. Table 2 shows the LBW distribution for the hospital-related variables: perinatal care level and baby-friendly status. The hospital perinatal care level indicates the level of services provided. *Basic care* provides services for low- to moderate-risk pregnancies. *Specialty care* offers services for the patients of obstetrics and neonatology at high risk. *Subspecialty care* provides all aspects of perinatal care, including intensive care and a range of continuously available subspecialty consultations as recommended. *Complex care* offers additional capabilities and considerable experience for newborn infants who are the most complex and critically ill, and pediatric medical and surgical specialty consultants are available 24 hours a day. We can then expect that the percentage of LBW increases with the perinatal care level. Specifically, the rate of LBW in complex care hospitals was 4 times higher than that in basic care hospitals. See the more detailed distribution in Table 2.

The hospital’s baby-friendly status indicates whether the hospital provides education to help families make informed decisions about breastfeeding, encourages patients to have skin-to-skin contact immediately after birth, and provides expert breastfeeding support during hospitalization at the time of delivery before discharge. Our data set showed that the percentage of LBW records in the baby-friendly hospital was higher than in the non–baby-friendly hospital given that higher-level hospitals serve the most complex cases and have more capacity to go through the baby-friendly designation process.

The *P* value from the chi-square test reported in Table 2 showed a statistically significant association between the categories for each hospital-related variable and LBW (all *P*<.001).

**Table 2:** Difference between low birthweight (LBW) and non-LBW groups in hospital-related variables (N=255,467).

Hospital-related variables			All records							LBW versus non-LBW, odds ratio (95% CI)
			LBW group (n=23,019), n (%)		Non-LBW group (n=232,448), n (%)		*P* value			
**Hospital perinatal care level**										
	Basic care	1715 (7.45)		33,183 (14.28)		<.001		0.45 (0.38-0.55)		
	Specialty care	8139 (35.36)		110,373 (47.48)		<.001		0.65 (0.54-0.78)		
	Subspecialty care	12,657 (54.99)		86,188 (37.08)		<.001		1.29 (1.07-1.55)		
	Complex care	380 (1.65)		1579 (0.68)		<.001		2.12 (1.71-2.62)		
	Unknown	128 (0.56)		1125 (0.48)		<.001		—^a^		
**Hospital baby-friendly status**										
	Yes	12,371 (53.74)		93,883 (40.39)		<.001		1.72 (1.67-1.76)		
	No	10,648 (46.26)		138,565 (59.61)		<.001		—		

^a^Reference group for odds ratio.

#### Vulnerability Index on LBW

Our data set also included different SVI indicators measured at the census tract level based on the mother’s residence. The SVI score indicates the potential adverse effects on communities caused by external stresses on human health, such as natural or human-caused disasters [43]. The CDC assigned an SVI score for each of the census tracts based on the social factors for each SVI variable from US Census data. The social factors for each SVI variable are as follows: (1) *SVI_SocioEconomic*: below poverty, unemployed, low income, or no high school diploma; (2) *SVI_HouseholdComp*: aged ≥65 years, aged ≤17 years, aged >5 years with a disability, or single-parent households; (3) *SVI_MinorityStatus*: minority race or insufficient English language level; and (4) *SVI_HousingType*: multiunit structures, mobile homes, crowding, no vehicle, or group quarters.

The range of SVI scores is from 0 to 1. A higher SVI score means that the community is more vulnerable and less resilient, requiring more resources to thrive. A lower SVI score indicates that the community is less vulnerable to external stress. Table 3 shows the distribution of each SVI indicator in our data set for the LBW and non-LBW groups.

As shown in Table 3, the mean values for all SVI variables in the LBW group were higher than those of the non-LBW group, which means that the communities are more vulnerable and populated with patients susceptible to LBW.

**Table 3:** Difference between low birthweight (LBW) and non-LBW groups in social vulnerability index (SVI)–related variables (N=255,467).

SVI-related variables	All records, mean (SD)		
	LBW group (n=23,019)	Non-LBW group (n=232,448)	*P* value
SVI_SocioEconomic	0.421 (0.28)	0.370 (0.28)	<.001
SVI_HouseholdComp	0.486 (0.27)	0.447 (0.27)	<.001
SVI_MinorityStatus	0.603 (0.27)	0.581 (0.27)	<.001
SVI_HousingType	0.566 (0.27)	0.529 (0.27)	<.001
SVI_TotalScore	0.421 (0.28)	0.370 (0.28)	<.001

#### Patient-Related Variables on LBW

Table 4 shows the distribution of the LBW records for each pregnancy-related variable. Pregnant patient–related variables included the patients’ demographics and preexisting conditions. In addition, in our data set, patients’ preexisting composite chronic and behavioral health conditions that are the most relevant to poor birth outcomes were also included. These were defined as having an inpatient stay or ED visit with a primary or secondary code on the record for one of these conditions. Chronic conditions included cardiovascular diseases, hypertension, diabetes, and obesity, and the behavioral health variables covered substance abuse and mental health. We also included a variable named "disease profile," which indicates the patients' chronic and behavioral health conditions. The percentage of LBW records indicates the proportion of LBW records among all delivery records for a specific category. We found that the rates of LBW records in patients aged 10-19 years and 30-54 years were higher than those in other age groups. The percentage of LBW in patients self-identifying as non-Hispanic Black individuals was substantially higher than that in other racial groups. Patients who enrolled in the Medicaid program had a higher percentage of LBW than those with other payment sources. Regarding birth history and plurality indicators, first-time pregnant patients and those carrying multiple fetuses were more likely to have LBW than other pregnant adults. Especially for the number of fetuses delivered, the percentage of LBW for mothers with multiple fetuses was 9 times higher than for those carrying a singleton. For all preexisting conditions, the LBW percentage for mothers with preexisting conditions was higher than for those without them. We also performed the chi-square test between each input variable and the target variable. The results showed that the difference between the categories for each variable in the LBW group was statistically significant (all *P*<.001).

Race and payment source are also 2 frequently cited key indicators to identify health disparities. To investigate the impact of race and payment source on LBW records, we studied how LBW distribution was affected by these 2 variables (Table 5). In our data set, 32.33% (82,592/255,467) of our records were from patients who self-identified as non-Hispanic Black individuals, the largest racial group. The percentage of LBW in self-identified non-Hispanic Black patients (11,400/82,592, 13.8%) was more than twice that of patients who self-identified as non-Hispanic White (9514/142,682, 6.67%) or Hispanic (1565/23,938, 6.54%) individuals. Regarding payment source, 62.2% (159,019/255,467) of patients were enrolled in Medicaid, which was the most common payment source in our data set. Those who were enrolled in Medicaid were more likely to have LBW (16,908/159,019, 10.63%) than patients with other payment sources (6111/96,448, 6.34%). As shown in Table 5, self-identified non-Hispanic Black patients who were enrolled in Medicaid had the highest chance of LBW compared with other groups. Approximately 14.08% (9921/70,451) of the self-identified non-Hispanic Black patients who were enrolled in the Medicaid program had LBW, which was substantially higher than that of other ethnic groups (Table 5).

In addition to statistically different factors on LBW distribution, we used all predelivery variables from different perspectives, including hospital, maternal demographics, and preexisting conditions to predict LBW using ML models.

**Table 4:** Difference between low birthweight (LBW) and non-LBW groups in pregnancy-related variables (N=255,467).

Pregnant patient–related variables		All records				LBW versus non-LBW, odds ratio (95% CI)
		LBW group (n=23,019), n (%)	Non-LBW group (n=232,448), n (%)	*P* value		
**Age group (years)**						
	10-19	1779 (7.73)	15,093 (6.49)	<.001	—^a^	
	20-24	5798 (25.19)	54,676 (23.52)	<.001	0.90 (0.85-0.92)	
	25-29	6505 (28.26)	71,556 (30.78)	<.001	0.77 (0.73-0.82)	
	30-34	5278 (22.93)	58,967 (25.37)	<.001	0.76 (0.72-0.80)	
	35-54	3659 (15.9)	32,156 (13.83)	<.001	0.97 (0.91-1.03)	
**Race**						
	Non-Hispanic White	9514 (41.33)	133,168 (57.29)	<.001	0.76 (0.69-0.83)	
	Non-Hispanic Black	11,400 (49.52)	71,192 (30.63)	<.001	1.70 (1.55-1.86)	
	Hispanic	1565 (6.8)	22,373 (9.62)	<.001	0.74 (0.67-0.82)	
	Other non-Hispanic races	540 (2.35)	5715 (2.46)	<.001	—	
**Payment source**						
	Private	4715 (20.48)	72,929 (31.37)	<.001	0.79 (0.74-0.84)	
	Medicaid	16,908 (73.45)	142,111 (61.14)	<.001	1.45 (1.36-1.54)	
	Uninsured	218 (0.95)	3084 (1.33)	<.001	0.86 (0.74-1.00)	
	Other	1178 (5.12)	14,324 (6.16)	<.001	—	
**First-time pregnancy**						
	Yes	9941 (43.19)	88,788 (38.2)	<.001	1.23 (1.20-1.26)	
	No	13,078 (56.81)	143,660 (61.8)	<.001	—	
**Number of fetuses delivered**						
	Singleton	19,907 (86.48)	231,046 (99.4)	<.001	—	
	Multiple	3112 (13.52)	1402 (0.6)	<.001	25.76 (24.14-27.48)	
**Cardiovascular disease (previous 12 months)**						
	Yes	1946 (8.45)	6237 (2.68)	<.001	3.35 (3.18-3.53)	
	No or unknown	21,073 (91.55)	226,211 (97.32)	<.001	—	
**Hypertension (previous 12 months)**						
	Yes	1723 (7.49)	4711 (2.03)	<.001	3.91 (3.70-4.14)	
	No or unknown	21,296 (92.51)	227,737 (97.97)	<.001	—	
**Hypertension in pregnancy (previous 12 months)**						
	Yes	1315 (5.71)	4234 (1.82)	<.001	3.27 (3.07-3.48)	
	No or unknown	21,704 (94.29)	228,214 (98.18)	<.001	—	
**Diabetes (previous 12 months)**						
	Yes	605 (2.6)	2417 (1.04)	<.001	2.57 (2.35-2.81)	
	No or unknown	22,414 (97.37)	230,031 (98.96)	<.001	—	
**Gestational diabetes (previous 12 months)**						
	Yes	330 (1.4)	1941 (0.84)	<.001	1.73 (1.54-1.94)	
	No or unknown	22,689 (98.57)	230,507 (99.16)	<.001	—	
**Obesity (previous 12 months)**						
	Yes	1038 (4.51)	4269 (1.84)	<.001	2.53 (2.36-2.71)	
	No or unknown	21,981 (95.49)	228,179 (98.16)	<.001	—	
**Substance abuse (previous 12 months)**						
	Yes	3045 (13.23)	16,081 (6.92)	<.001	2.05 (1.97-2.14)	
	No or unknown	19,974 (86.77)	216,367 (93.08)	<.001	—	
**Mental health (previous 12 months)**						
	Yes	3873 (16.83)	21,435 (9.22)	<.001	1.99 (1.92-2.07)	
	No or unknown	19,146 (83.17)	211,013 (90.78)	<.001	—	
**ICU** ^b^ **stay (previous 12 months)**						
	Yes	107 (0.5)	335 (0.1)	<.001	3.24 (2.61-4.03)	
	No or unknown	22,912 (99.54)	232,113 (99.86)	<.001	—	
**Pre-eclampsia (previous 12 months)**						
	Yes	317 (1.4)	252 (0.1)	<.001	12.87 (10.90-15.19)	
	No or unknown	22,702 (98.62)	232,196 (99.89)	<.001	—	
**Chronic conditions (previous 12 months)**						
	Yes	2810 (12.21)	10,867 (4.68)	<.001	2.84 (2.72-2.96)	
	No or unknown	20,209 (87.79)	221,581 (95.32)	<.001	—	
**Behavioral health (previous 12 months)**						
	Yes	4011 (17.42)	22,283 (9.59)	<.001	1.99 (1.92-2.07)	
	No or unknown	19,008 (82.58)	210,165 (90.41)	<.001	—	
**Disease profile (previous 12 months)**						
	Chronic only	1741 (7.56)	7147 (3.07)	<.001	0.85 (0.78-0.92)	
	Behavioral health only	2942 (12.78)	18,563 (7.99)	<.001	0.55 (0.51-0.60)	
	Both chronic and behavioral health	1069 (4.64)	3720 (1.60)	<.001	—	
	Either or both chronic and behavioral health	17,267 (75.01)	203,018 (87.34)	<.001	2.30 (0.28-0.32)	

^a^Reference group for odds ratio.

^b^ICU: intensive care unit.

**Table 5:** Percentage of low birthweight by race and payment source.

Race	Private, n/N (%)	Medicaid, n/N (%)	Uninsured, n/N (%)	Other, n/N (%)
Non-Hispanic White	3344/63,857 (5.24)	5532/67,682 (8.17)	113/1971 (5.7)	525/9172 (5.7)
Non-Hispanic Black	950/8198 (11.6)	9921/70,451 (14.08)	45/371 (12)	484/3572 (13.5)
Hispanic	177/2800 (6.3)	1202/18,065 (6.65)	50/804 (6)	136/2269 (6)
Other non-Hispanic races	244/2789 (8.7)	253/2821 (9)	10/156 (6)	33/489 (7)

### Model Performance of LBW Prediction

#### Model Performance on the Original Clean Data

First, we compared the model performance based on the original clean data set without data rebalancing. As shown in Table 6, all ML models achieved >85% average overall accuracy, whereas the average recall score for the LBW group was <0.4.

NB had the best performance for the LBW group, with a recall score of 0.35. The high accuracy could be misleading as the data set was highly imbalanced and the model tended to be more biased toward the majority class to achieve higher accuracy. The issue of imbalanced data is also a frequent problem in public health as the study target is often the minority class. In our study, because of the focus on predicting the minority class (LBW), it was crucial to adequately handle the imbalanced data and achieve good performance in the minority group.

**Table 6:** Model performance comparison on the original imbalanced dataset.

Model	Average accuracy	Average precision	Average recall	Average *F*_1_-score	Average AUROC^a^
LR^b^	0.92	0.69	0.14	0.24	0.57
NB^c^	0.85	0.25	0.35	0.29	0.62
RF^d^	0.90	0.42	0.15	0.22	0.57
XGBoost^e^	0.92	0.70	0.15	0.24	0.57
AdaBoost^f^	0.92	0.70	0.14	0.23	0.57
MLP^g^	0.92	0.67	0.15	0.24	0.57
Sequential ANN^h^	0.92	0.64	0.14	0.23	0.57

^a^AUROC: area under the receiver operating characteristic curve.

^b^LR: logistic regression.

^c^NB: naive Bayes.

^d^RF: random forest.

^e^XGBoost: extreme gradient boosting.

^f^AdaBoost: adaptive boosting.

^g^MLP: multilayer perceptron.

^h^ANN: artificial neural network.

#### Model Performance on Rebalanced Data

Table 6 shows that all applied prediction models achieved good overall accuracy; however, the recall scores were very low, meaning that the models could not accurately identify most LBW cases in the testing set. These models tend to predict the case as non-LBW to obtain higher accuracy from the highly imbalanced data set. To reduce the impact of data imbalance on our ML prediction task, we applied undersampling, random oversampling, SMOTE, and class weight adjustment methods to balance the LBW and non-LBW groups on the training set and then evaluated the models on the original testing set. In Table 7, we compared the ML models’ performance using each rebalancing method.

Using the undersampling technique, the recall scores of the predictive models showed substantial improvement. Among them, RF achieved the highest recall score of 0.62, and the AUROC score also increased from 0.57 to 0.63. Except for NB, all the other models achieved a recall score of >0.60, and MLP had the highest AUROC score of 0.72, which was a substantial improvement over its previous score of 0.57. Although NB obtained the lowest recall score, it had the highest precision score.

We evaluated the models’ performance after applying the random oversampling technique to the training set. LR, XGBoost, and AdaBoost achieved the highest recall score of 0.61 and the highest AUROC score of 0.67. The recall scores of the NB and RF models were noticeably lower than those of the other models.

When using another oversampling technique, SMOTE, the highest-performing model was LR, with a recall score of 0.58 and an AUROC score of 0.66. However, the other models’ performances with the SMOTE method were considerably lower than that of the LR model. In comparison with the other rebalancing techniques, the models trained on the SMOTE-rebalanced data set had the lowest performance.

Upon cross-comparing the recall scores of the models, we discovered that the weight rebalancing method was not appropriate for all models. XGBoost achieved the highest recall score of 0.70 and the highest AUROC score of 0.67 among all the other models trained using weight rebalancing. This recall score was also the highest among all the other models trained using other rebalancing methods. However, the performances of other models, such as RF and AdaBoost, were similar to the performances of those trained using the original imbalanced data.

In summary, the recall score substantially improved from 0.15 to 0.70 using the weight rebalancing technique and XGBoost. The composite measurement AUROC score increased from 0.57 to 0.67 using all rebalancing methods except for SMOTE. XGBoost outperformed or was at least comparable with all other ML models consistently for different rebalancing methods except for SMOTE. Furthermore, most of the applied models demonstrated improved performance using the random undersampling or random oversampling techniques. However, SMOTE was the least effective rebalancing method based on the models’ performance.

To further validate the methodology that we applied to the LBW prediction task, we applied the same techniques to the ELBW prediction task and cross-compared the performance before and after the rebalanced training set using 5-fold cross-validation. The results are presented in Table 8.

**Table 7:** Model performance comparison—rebalanced data.

Method and model		Average accuracy	Average precision	Average recall	Average *F*_1_-score	Average AUROC^a^
**Random undersampling**						
	LR^b^	0.72	0.18	0.61	0.28	0.67
	NB^c^	0.83	0.22	0.39	0.28	0.63
	RF^d^	0.65	0.15	0.62	0.24	0.63
	XGBoost^e^	0.71	0.18	0.61	0.28	0.67
	AdaBoost^f^	0.72	0.18	0.61	0.28	0.67
	MLP^g^	0.72	0.18	0.60	0.28	0.72
	Sequential ANN^h^	0.69	0.17	0.61	0.26	0.65
**Random oversampling**						
	LR	0.72	0.18	0.61	0.28	0.67
	NB	0.83	0.23	0.38	0.28	0.63
	RF	0.83	0.19	0.26	0.22	0.57
	XGBoost	0.72	0.18	0.61	0.28	0.67
	AdaBoost	0.72	0.18	0.61	0.28	0.67
	MLP	0.72	0.18	0.60	0.28	0.67
	Sequential ANN	0.69	0.17	0.61	0.26	0.65
**SMOTE^i^**						
	LR	0.73	0.18	0.58	0.28	0.66
	NB	0.82	0.22	0.39	0.28	0.63
	RF	0.89	0.31	0.22	0.26	0.59
	XGBoost	0.89	0.31	0.22	0.26	0.59
	AdaBoost	0.85	0.27	0.36	0.31	0.63
	MLP	0.84	0.25	0.38	0.30	0.63
	Sequential ANN	0.69	0.16	0.55	0.24	0.63
**Weight rebalancing**						
	LR	0.72	0.18	0.61	0.28	0.67
	NB	0.82	0.22	0.39	0.28	0.63
	RF	0.87	0.21	0.18	0.20	0.56
	*XGBoost* ^j^	*0.64*	*0.16*	*0.70*	*0.26*	*0.67*
	AdaBoost	0.81	0.15	0.24	0.18	0.55

^a^AUROC: area under the receiver operating characteristic curve.

^b^LR: logistic regression.

^c^NB: naive Bayes.

^d^RF: random forest.

^e^XGBoost: extreme gradient boosting.

^f^AdaBoost: adaptive boosting.

^g^MLP: multilayer perceptron.

^h^ANN: artificial neural network.

^i^SMOTE: synthetic minority oversampling technique.

^j^Model with the best performance is italicized.

**Table 8:** Model performance comparison for the extreme low birthweight prediction task.

Method and model		Average accuracy	Average precision	Average recall	Average *F*_1_-score	Average AUROC^a^
**Before rebalancing**						
	LR^b^	0.93	0.13	0.17	0.15	0.58
	NB^c^	0.87	0.05	0.45	0.09	0.66
	RF^d^	0.95	0.17	0.66	0.27	0.81
	XGBoost^e^	0.97	0.14	0.18	0.16	0.58
	AdaBoost^f^	0.97	0.14	0.18	0.16	0.58
	MLP^g^	0.97	0.14	0.18	0.16	0.58
	Sequential ANN^h^	0.98	0.15	0.02	0.04	0.51
**Random undersampling**						
	LR	0.74	0.04	0.80	0.08	0.77
	NB	0.87	0.05	0.46	0.10	0.67
	RF	0.74	0.04	0.77	0.08	0.75
	XGBoost	0.75	0.05	0.80	0.09	0.77
	AdaBoost	0.75	0.04	0.79	0.08	0.77
	MLP	0.71	0.04	0.72	0.07	0.72
	Sequential ANN	0.69	0.04	0.82	0.07	0.76
**Random oversampling**						
	LR	0.75	0.04	0.80	0.08	0.77
	NB	0.88	0.05	0.46	0.10	0.67
	RF	0.96	0.04	0.06	0.05	0.52
	XGBoost	0.76	0.05	0.78	0.09	0.77
	AdaBoost	0.76	0.05	0.78	0.09	0.77
	MLP	0.71	0.05	0.71	0.08	0.72
	Sequential ANN	0.80	0.04	0.57	0.08	0.69
**SMOTE^i^**						
	LR	0.80	0.05	0.68	0.09	0.74
	NB	0.82	0.05	0.59	0.09	0.71
	RF	0.98	0.11	0.06	0.08	0.53
	XGBoost	0.94	0.07	0.30	0.12	0.62
	AdaBoost	0.85	0.05	0.57	0.10	0.71
	MLP	0.94	0.06	0.21	0.09	0.58
	Sequential ANN	0.88	0.05	0.37	0.08	0.63
**Weight rebalancing**						
	LR	0.71	0.04	0.77	0.07	0.74
	NB	0.71	0.04	0.77	0.07	0.74
	RF	0.89	0.01	0.80	0.18	0.85
	XGBoost	0.61	0.03	0.85	0.06	0.61
	*AdaBoost* ^j^	*0.85*	*0.07*	*0.84*	*0.14*	*0.84*

^a^AUROC: area under the receiver operating characteristic curve.

^b^LR: logistic regression.

^c^NB: naive Bayes.

^d^RF: random forest.

^e^XGBoost: extreme gradient boosting.

^f^AdaBoost: adaptive boosting.

^g^MLP: multilayer perceptron.

^h^ANN: artificial neural network.

^i^SMOTE: synthetic minority oversampling technique.

^j^Model with the best performance is italicized.

RF achieved the best performance on the original imbalanced data set with a recall score of 0.66. After applying the data rebalancing methods, similar to the results for the LBW prediction task, XGBoost also achieved the highest recall score of 0.85 using weight rebalancing, but the AUROC score was only 0.61. The AdaBoost model achieved the second-highest recall score of 0.84 and the second-highest AUROC score of 0.84 using weight rebalancing. Therefore, the AdaBoost model using the weight rebalancing model was the best-performing model for the ELBW prediction task. Similar to the results of the LBW prediction task, SMOTE was the least effective rebalancing method compared with the other 3 methods applied.

Compared with the LBW prediction task, the ELBW prediction task achieved substantially higher recall and AUROC scores, both exceeding 0.80. The performance difference between these 2 tasks suggests that the distinction between the ELBW and non-ELBW groups is more apparent than the distinction between the LBW and non-LBW groups. In the following subsection, we will analyze the importance of features for the LBW prediction task to identify crucial patterns for recognizing the target group from the input features.

### Feature Importance Analysis

Feature importance analysis is the most common model interpretability method that provides the importance of each input feature on the target variable prediction. The feature importance score quantifies the features’ contribution to the model prediction output [65]. In this study, we selected the model with the best performance for each data rebalancing method to analyze the feature importance for the LBW prediction—the model performance considered by the recall scores. We compared the feature importance ranking before and after by applying the data rebalancing method and focusing on the features whose importance scores increased after the data rebalancing methods were applied. The increased recall score indicates that the prediction models achieved higher accuracy in the LBW group after being trained on the rebalanced data set. The increased importance scores indicate that these features contributed more to the accurate identification of LBW cases.

As SMOTE was the least effective rebalancing method compared with the other 3 methods applied, we did not consider SMOTE in the feature importance study. In the LBW prediction task, as the XGBoost model outperformed other models consistently using different rebalancing methods, we compared the top 20 features of the XGBoost models before and after rebalancing using undersampling, random oversampling, and weight rebalancing. As the features’ importance scores were calculated based on their contributions during classification, the high importance scores indicated that these features were more critical in identifying the LBW group.

**Figure 4:**
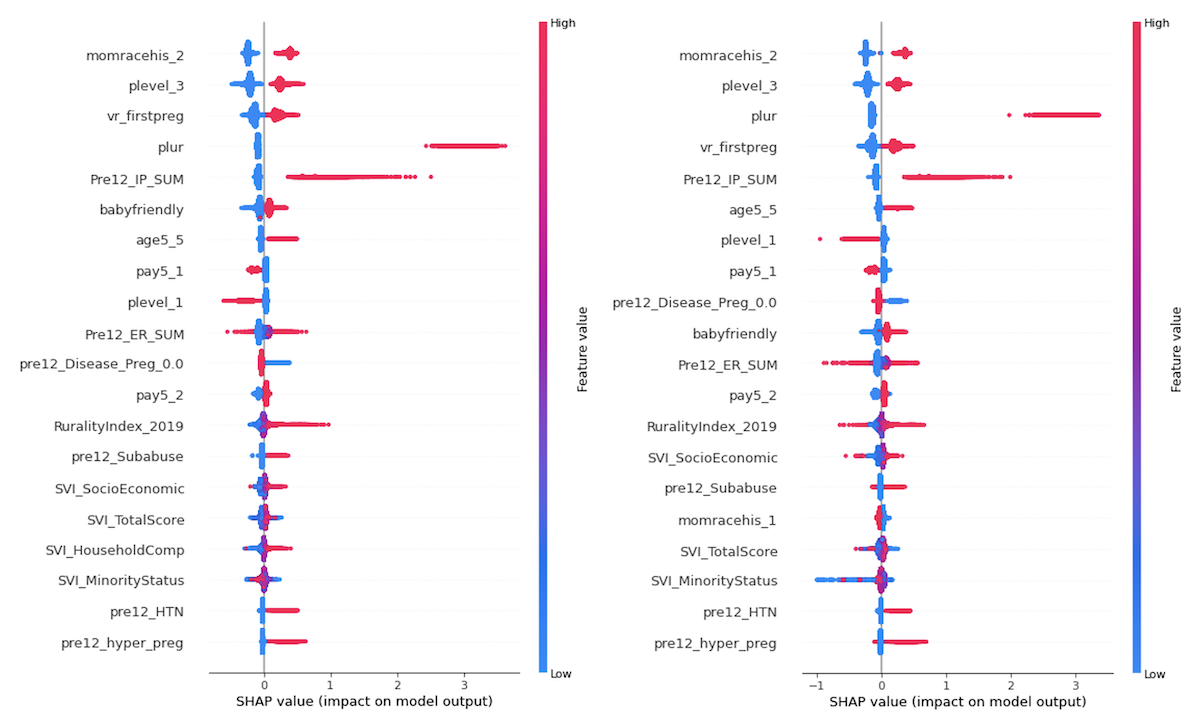
Top 20 feature importance ranking of extreme gradient boosting before (left) and after (right) using weight rebalancing. SHAP: Shapley additive explanations.

**Figure 5:**
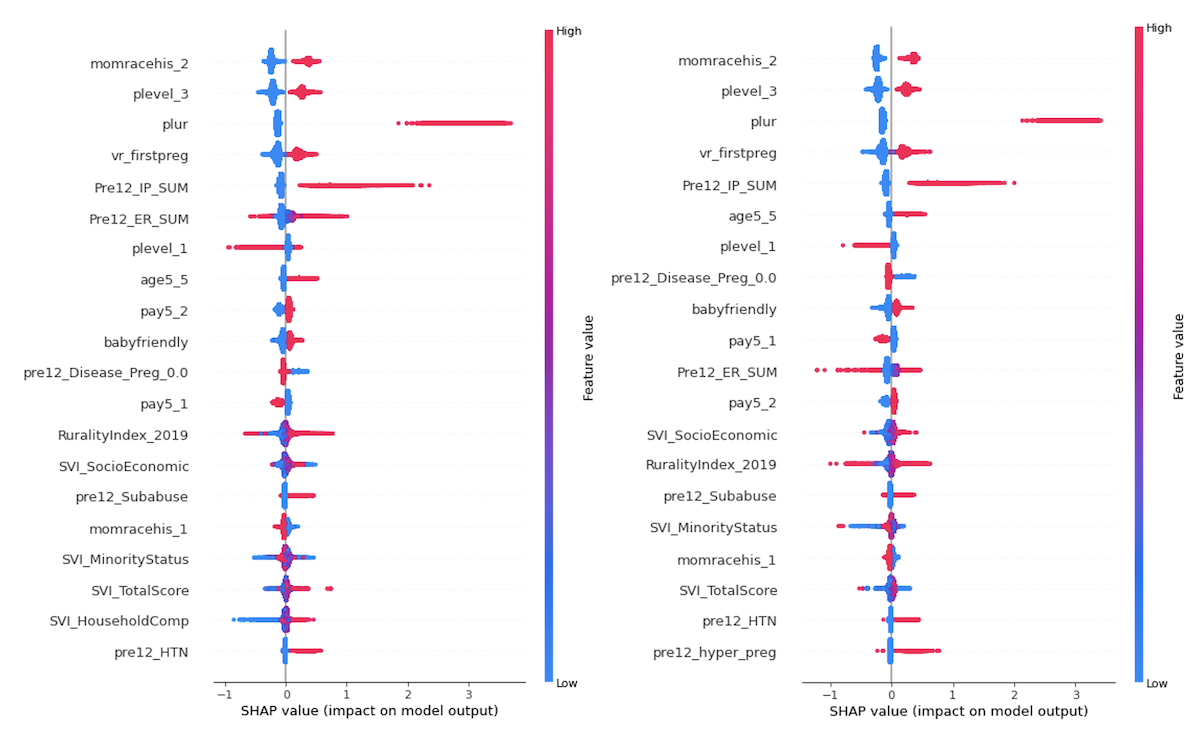
Feature importance comparison of extreme gradient boosting after random oversampling (left) and random undersampling (right). SHAP: Shapley additive explanations.

We conducted an analysis of these most important features using Shapley additive explanations (SHAP) values and generated the SHAP summary plots (Figures 4 and 5), which indicate the relationship between the value of the features and the impact on the prediction. The point colors from red to blue represent the feature values from high to low. The related SHAP value for each feature value represents this feature value’s contribution to the LBW prediction task.

As shown in Figure 4, mother’s race—non-Hispanic Black (*momracehis_2*), perinatal care level—subspecialty (*plevel_3*), number of fetuses delivered (*plur*), first-time pregnancy (*vr_firstpreg*), the sum of inpatient hospitalizations (*Pre12_IP_SUM*), mother’s age—35 to 54 years (*age5_5*), perinatal care level—basic (*plevel_1*), payment source—private (*pay5_1*), disease profile—either or both chronic and behavioral health (*pre12_Disease_Preg_0.0*), hospital baby-friendly status (*baby-friendly*), the sum of ED visits (*Pre12_ER_SUM*), payment source—Medicaid (*pay5_2*), rurality index (a measurement of rurality based on different indicators, such as population, extent of urbanized area, and distance to the nearest metropolitan area), SVI socioeconomic, preexisting substance abuse (*pre12_Subabuse*), SVI total score, SVI minority status, preexisting hypertension (*pre12_HTN*), and preexisting gestational hypertension (*pre12_hyper_preg*) were the most critical features before and after using weight rebalancing. Among these features, there were 3 social determinant indicators: SVI socioeconomic, SVI total score, and SVI minority status. A high importance score means that the social determinants based on patients’ residence were very important for LBW prediction.

Compared with the feature importance ranking before applying any rebalancing method, the feature mother’s race—non-Hispanic White (*momracehis_1*) became the top feature after weight rebalancing. Regarding the common features for both before and after weight rebalancing, the features of increased importance included the number of fetuses delivered, mother’s age—35 to 54 years, perinatal care level—basic (*plevel_1*), disease profile—either or both chronic and behavioral health, and SVI socioeconomic. The increased importance scores from the models that applied rebalancing methods indicate that these features contributed more to the accurate identification of LBW cases because the prediction models achieved higher recall score in the LBW group after being trained on the rebalanced data set. Comparing the rankings in Figure 4 based on the SHAP value, we find that a lower value of SVI minority status has a more substantial negative impact on the model output after weight rebalancing.

Figure 5 illustrates that the 20 most important features identified by XGBoost using both random under- or oversampling were identical to those displayed in Figure 4 and only differed slightly in ranking and SHAP value.

Among the binary categorical features, we found that the “Yes” category for the features, including mothers’ race—non-Hispanic Black, perinatal care level—subspecialty, first-time pregnancy, mothers’ age—35 to 54 years, hospital baby-friendly status, payment source—Medicaid, preexisting substance abuse, preexisting hypertension, and preexisting gestational hypertension, had substantially positive contributions to the model outcome. Conversely, the “No” category for perinatal care level—basic, payment source—private, disease profile—either or both chronic and behavioral health, and mother’s race—non-Hispanic White had a negative impact on the model outcome. We also found that a higher number of fetuses delivered and a higher sum of inpatient hospitalizations had a substantially positive contribution to the model outcome.

## Discussion

### Principal Findings

In this study, we analyzed 255,869 birth records from 2015 to 2021 in a southeastern state in the United States. Among all the data records, there were 9% (23,019/255,869) of LBW records. To identify the LBW records based on all selected predelivery features, we applied 7 classic ML models to classify the LBW and non-LBW records. To effectively handle the data imbalance problem, we explored 4 popular rebalancing methods and cross-compared the models’ performance for each method. Notably, we found that the highest recall score (0.70) was achieved by the XGBoost model after applying the weight rebalancing method to the model, indicating a considerable improvement compared with previous studies.

The findings of this study indicate that the SMOTE method was the least effective of the rebalancing methods. This could be because the SMOTE method generates synthetic samples in the minority class by interpolating between the feature values of the existing minority class samples, which can create unrealistic samples that do not accurately represent the true distribution of the minority class. This can lead to overfitting and reduced model generalization. Furthermore, the SMOTE method may increase the overlap between the minority and majority classes, resulting in a more challenging classification problem. In some instances, SMOTE may even introduce noise into the data set, leading to a reduction in model performance.

We then performed the feature importance analysis based on the SHAP value for the best-performing model in random undersampling, random oversampling, and weight rebalancing—XGBoost. We compared the top 20 feature importance ranking using these 3 rebalancing methods and focused on the common important features. We found that the 20 most important features identified by XGBoost using those 3 rebalancing methods were identical. The common features were then analyzed using SHAP values, which indicated that certain binary categorical features such as patient race—non-Hispanic Black, perinatal care level—subspecialty, and first-time pregnancy have substantially positive contributions to the model output if these binary features equal to “Yes (1).” In addition, a higher number of fetuses delivered and a higher sum of inpatient hospitalizations had a substantially positive contribution to the model output.

On the basis of the frequency analysis, we found that the LBW risk in adolescent and advanced maternal age groups was higher than in other age groups. This finding is consistent with previous research [66]. On the basis of the patients’ race, we found that the connection between LBW and non-Hispanic Black patients was substantially higher than that in the other racial groups. Previous studies conducted in other US states have also found that the LBW risk in non-Hispanic Black mother groups was higher than that in other racial groups [67-69], suggesting the negative impacts of experiencing racism and health disparities [70].

One of our aims was to precisely identify patients at the greatest risk of having a baby with LBW by providing evidence-based research to inform changes to Medicaid policy that would support early intervention for these patients. In our model, we observed that patients who were enrolled in the Medicaid program did have a higher chance of having a baby with LBW than those who used other payment sources. Another critical health disparity–related finding was that non-Hispanic Black patients who were enrolled in Medicaid programs had a substantially higher LBW risk than other groups, noting the great importance of early holistic care services for these patients. Given the disproportionate burden to the Medicaid program of treating patients with high-cost deliveries, including but not limited to the care of their newborns receiving neonatal intensive care services, Medicaid investments addressing the social determinants of health identified in our models present an opportunity to improve maternal and child health outcomes, reduce disparities, and save state dollars.

Our study also indicated that first-time pregnant patients were more likely to have LBW than other experienced mothers, consistent with a previous study conducted outside the United States [71]. Regarding plurality indicators, pregnant women carrying multiple babies had a substantially higher LBW risk than other mothers, confirming a national report on the contribution of singletons, twins, and triplets to LBW [72]. The risk of LBW in patients with any preexisting condition, including cardiovascular disease, hypertension, gestational hypertension, diabetes, gestational diabetes, obesity, substance abuse, mental health, and pre-eclampsia, was 2 times higher than in those without preexisting conditions, which is consistent with other study findings [73-75]. Our feature importance analysis results also aligned well with our statistical analysis and previous findings. The preexisting condition feature, that is, disease profile—either or both chronic and behavioral health conditions—was identified as an important feature in all 3 models as pregnant patients with preexisting health conditions contribute more to differentiating the LBW and non-LBW records. Regarding the social determinants, we found that the residence of the patients with an LBW birth was more vulnerable than that of patients with a non-LBW birth.

The important features found in our study can be used by clinicians to identify the pregnant patients at the highest risk at an early stage of their pregnancy through early prenatal care screening tools. These identified patients could be advised to obtain access to additional support for a healthy pregnancy through group prenatal care and doula and community health worker services, as well as through primary and specialist treatment for the management of comorbidities of high feature importance. As fetal growth and development are a dynamic process with many uncertainties, it is important to note that predicting LBW using only features available in the early stages of pregnancy and before any further examinations may not always be accurate, thus leading to unnecessary stress and anxiety for the expectant parents. As such, health care professionals and providers should be cautious in considering the potential benefits and drawbacks of sharing such information with patients and making decisions in the best interest of the patient’s overall health and well-being.

### Limitations and Future Directions

This study had several limitations. As our data were collected based on the linkage of vital statistics birth records with ED visits and inpatient hospital record data, there are a certain number of missing values in different variables owing to the mother not meeting the inclusion criteria for a measure or having no visits. These missing values could introduce different biases to our data after we cleaned the data set. Moreover, the preexisting conditions and other categorical variables in our data set were aggregated and categorized into broad groups based on the agency information disclosure policy. These broad categories directly lead to data records with high similarity in our data set and, thus, impose obstacles to effectively improve ML model performance.

Another limitation involves information asymmetry. During pregnancy, patients may need to visit an obstetrics physician regularly for different diagnoses. If the diagnosis results indicate that the mother has a higher risk of adverse birth outcomes, the physician will recommend the pregnant adult for early intervention and adjust the treatment schedule to make a positive impact on the birth outcome. Unfortunately, we did not have outpatient diagnosis information accessible to us in this study, limiting our ability to provide additional valuable insights.

The lack of model generalization experiments was also a limitation of this study. The model generalization capability indicates the model performance on the data set that it was not specifically trained on. Thus, it is an important aspect of ML models as it helps improve the reliability and robustness of the model. However, the complexity and volume of data from various sources, combined with privacy and security concerns, can make the health care data collection process slow and challenging. Thus, the results from the benchmarking models are only focused on the specific data that we used in this study rather than preparing different data sets and producing the experiments for model generalizability.

For future studies, we will work with our data providers to extract more information to differentiate the identical records in the current data set, focusing on incorrectly predicted records, and introduce novel classification methods to improve model performance. In contrast, we will develop a deep neural network model with an optimized architecture and loss function to further improve the performance of LBW prediction for a highly imbalanced data set. Furthermore, we will also focus on developing a transfer learning model to exploit the external data set to enrich the information and improve the model performance for the LBW group. Moreover, we will also use the external data set to examine the benchmarking models’ generalizability to improve the models’ reliability and robustness. We are also working with our data providers to include additional features, such as adequacy of prenatal care, which will address the data limitation regarding the lack of outpatient records.

As a result, this study contributes substantially to the current perinatal care literature by being the first study to systematically apply both ML and rebalancing methods to more effectively handle extremely imbalanced data set issues for higher quality of prediction. In doing so, this study provides a set of benchmarking results that can be used to adapt health care policies, particularly for Medicaid, and support clinicians with early prenatal interventions for pregnant patients likely at the highest risk of LBW.

## Appendix

Multimedia Appendix 1 Model evaluation matrix.

## Abbreviations

AdaBoost: adaptive boosting
ANN: artificial neural network
AUROC: area under the receiver operating characteristic curve
CDC: Centers for Disease Control and Prevention
ED: emergency department
ELBW: extreme low birthweight
ICD-10: International Classification of Diseases, 10th Revision
LBW: low birthweight
LR: logistic regression
ML: machine learning
MLP: multilayer perceptron
NB: naive Bayes
OR: odds ratio
RF: random forest
SHAP: Shapley additive explanations
SMOTE: synthetic minority oversampling technique
SVI: social vulnerability index
SVM: support vector machine
UB: uniform billing
XGBoost: extreme gradient boosting

## Data Availability

Access to the data set analyzed in this study is not available to researchers because of Health Insurance Portability and Accountability Act and institutional review board constraints.
