# Supplementary material for: Issue of Data Imbalance on Low Birthweight Baby Outcomes Prediction and Associated Risk Factors Identification: Establishment of Benchmarking Key Machine Learning Models With Data Rebalancing Strategies
Source: J Med Internet Res. 2023 May 31;25:e44081. doi: 10.2196/44081 (PMC10267797; doi:10.2196/44081)
Supplement: Multimedia Appendix 1 [file jmir_v25i1e44081_app1.docx]

## **Appendix**

A1. List of Abbreviations

| Abbreviation | Full Name | Category |
| --- | --- | --- |
| LBW | Low Birthweight | Target Name |
| ML | Machine Learning | Study Method |
| LR | Logistic Regression | Classifier |
| RF | Random Forest | Classifier |
| MLP | Multilayer Perceptron | Classifier |
| SVM | Support vector machine | Classifier |
| KNN | K-nearest neighbors | Classifier |
| NN | Neural Network | Classifier |
| CT | Classification Tree | Classifier |
| DT | Decision Tree | Classifier |
| BF Tree | Best-First Tree | Classifier |
| REP Tree | Reduced Error Pruning Tree | Classifier |
| SMOTE | Synthetic Minority Oversampling Technique | Data Rebalance Method |
| SVI | Social Vulnerability Index | Feature Name |

A2. Model evaluation matrix

To evaluate the performance of each classification model, we employed confusion matrix for each model [55]. There are four different numbers in the matrix:

1. True Positive (TP): The sample is LBW, and the prediction result is also LBW.
2. False Negative (FN): The sample is LBW, but the model predicted it as non-LBW.
3. False Positive (FP): The sample is non-LBW, but the model predicted it as LBW.
4. True Negative (TN): The sample is non-LBW, and the prediction result is also non-LBW.

We employed five different metrics widely used for measuring classification performance including *Accuracy* (Equation 2), *Precision* (Equation 3), *Recall* (Equation 4), *F*_1_-score (Equation 5), and Area Under Receiver Operating Characteristic (AUROC) curve. The AUROC curve plots based on the Recall and False Positive Rate (FPR) (Equation 6). The *accuracy* score shows the ratio of all correctly predicted samples over the total samples. The *precision* measures the model's capability of not predicting the non-LBW cases as the LBW cases. The *Recall* score indicates the rate of correctly predicted LBW cases among all LBW cases. *F1-score* measures the harmonic mean of the Precision and Recall. And the *AUROC* is a measurement of the model accuracy.

| Accuracy = | TP + TN | (2) |
| --- | --- | --- |
|  | TP + FN + FP + TN |  |

| Precision = | TP | (3) |
| --- | --- | --- |
|  | TP + FP |  |

| Recall = | TP | (4) |
| --- | --- | --- |
|  | TP + FN |  |

| *F*_1_-score = | 2 * Precision * Recall | (5) |
| --- | --- | --- |
|  | Precision + Recall |  |

| FPR = | FP | (6) |
| --- | --- | --- |
|  | TN + FP |  |
